# An enhancer variant associated with breast cancer susceptibility in Black women regulates *TNFSF10* expression and antitumor immunity in triple-negative breast cancer

**DOI:** 10.1093/hmg/ddac168

**Published:** 2022-08-05

**Authors:** Yoo Jane Han, Jing Zhang, Ashley Hardeman, Margaret Liu, Olga Karginova, Roger Romero, Galina F Khramtsova, Yonglan Zheng, Dezheng Huo, Olufunmilayo I Olopade

**Affiliations:** Section of Hematology/Oncology & Center for Clinical Cancer Genetics, Department of Medicine, University of Chicago, Chicago, IL 60637, USA; Section of Hematology/Oncology & Center for Clinical Cancer Genetics, Department of Medicine, University of Chicago, Chicago, IL 60637, USA; Section of Hematology/Oncology & Center for Clinical Cancer Genetics, Department of Medicine, University of Chicago, Chicago, IL 60637, USA; Section of Hematology/Oncology & Center for Clinical Cancer Genetics, Department of Medicine, University of Chicago, Chicago, IL 60637, USA; Section of Hematology/Oncology & Center for Clinical Cancer Genetics, Department of Medicine, University of Chicago, Chicago, IL 60637, USA; Section of Hematology/Oncology & Center for Clinical Cancer Genetics, Department of Medicine, University of Chicago, Chicago, IL 60637, USA; Section of Hematology/Oncology & Center for Clinical Cancer Genetics, Department of Medicine, University of Chicago, Chicago, IL 60637, USA; Section of Hematology/Oncology & Center for Clinical Cancer Genetics, Department of Medicine, University of Chicago, Chicago, IL 60637, USA; Department of Public Health Sciences, University of Chicago, Chicago, IL 60637, USA; Section of Hematology/Oncology & Center for Clinical Cancer Genetics, Department of Medicine, University of Chicago, Chicago, IL 60637, USA

## Abstract

Women of African ancestry have the highest mortality from triple-negative breast cancer (TNBC) of all racial groups. To understand the genomic basis of breast cancer in the populations, we previously conducted genome-wide association studies and identified single nucleotide polymorphisms (SNPs) associated with breast cancer in Black women. In this study, we investigated the functional significance of the top associated SNP rs13074711. We found the SNP served as an enhancer variant and regulated *TNFSF10* (*TRAIL*) expression in TNBC cells, with a significant association between the SNP genotype and *TNFSF10* expression in breast tumors. Mechanistically, rs13074711 modulated the binding activity of c-MYB at the motif and thereby controlled *TNFSF10* expression. Interestingly, *TNFSF10* expression in many cancers was consistently lower in African Americans compared with European Americans. Furthermore, *TNFSF10* expression in TNBC was significantly correlated with the expression of antiviral immune genes and was regulated by type I interferons (IFNs). Accordingly, loss of *TNFSF10* resulted in a profound decrease in apoptosis of TNBC cells in response to type I IFNs and poly(I:C), a synthetic analogue of double stranded virus. Lastly, in a syngeneic mouse model of breast cancer, *TNFSF10*-deficiency in breast tumors decreased tumor-infiltrated CD4+ and CD8+ T cell quantities. Collectively, our results suggested that TNFSF10 plays an important role in the regulation of antiviral immune responses in TNBC, and the expression is in part regulated by a genetic variant associated with breast cancer in Black women. Our results underscore the important contributions of genetic variants to immune defense mechanisms.

## Introduction

Black women of African Ancestry in the US experience the highest mortality rates from breast cancer despite having slightly lower incidences of the disease compared with women of other ethnic groups. There has been marked improvement in access to mammographic screening, which now exceeds or equals screening rates in non-Hispanic Whites (1). Although improved access has led to equal rates in the diagnosis of ductal carcinoma *in situ*, there has been no decline in the proportion of African Americans (AA) women diagnosed with triple-negative breast cancer (TNBC) of advanced stages (2,3). This indicates that, despite having access to proper screening and standard treatments (4), women of African descent are more likely to be diagnosed with aggressive types of breast cancer, have inferior therapeutic outcomes, poorer responsiveness to chemotherapy and more therapy-induced toxicity (3,5–8).

To understand the genomic basis of health disparities and to accelerate progress in the field, we conducted a large scale genome-wide association studies (GWAS) using combined samples of breast cancer cases and controls in Africa, Caribbean countries and the USA (4673 cases and 4774 controls of African ancestry in the discovery phase and 1984 cases and 2939 controls in the validation phase). We identified single nucleotide polymorphisms (SNPs) associated with estrogen receptor (ER)-negative breast cancer in women of African descent (5). The top SNP, rs13074711, is located at 3q26, $\sim$26.5 kb upstream of the *TNFSF10* gene. Intriguingly, this region includes enhancer marks as well as an open chromatin structure, suggesting a potential role for the SNP (and linked SNPs) to serve as an enhancer that regulates *TNFSF10* gene expression.

TNFSF10 (also well-known as TRAIL), a member of the tumor necrosis factor (TNF) superfamily, is a homotrimeric type II transmembrane ligand that preferentially induces apoptosis in tumor cells or transformed cells, but is not toxic to normal cells (6,7). Interaction of TNFSF10 with its receptors recruits the adaptor molecule FADD, activates caspases and degrades cellular components, ultimately leading to apoptosis of cancer cells. Interestingly, recent evidence suggests a significant role for TNFSF10/TRAIL in regulating antitumor immunity in cancer cells and the tumor microenvironment (TME) (8). TRAIL promotes granzyme B expression in cytotoxic T-cells, yet can also stimulate proliferation of regulatory T cells and M2 macrophages. These data suggest that TNFSF10 not only promotes cell death but also regulates immune cell function and proliferation.

Tumor immunity and immunotherapy have become increasingly important in treatment strategies for a variety of malignancies including advanced TNBC (9,10). The efficacy of cancer immunotherapy appears to depend on the host immune system recognizing and eliminating cancer cells (11). Increasing evidence supports a positive correlation between the presence of host antitumor immune responses and favorable patient outcomes for many cancers (12–15). As an example, tumors with a high density of tumor-infiltrating lymphoid cells (TILs) in the TME are more likely to respond to immune checkpoint inhibitors, whereas those with low or no TILs are less likely to respond to the inhibitors (16–18). Thus, interventions that promote antitumor immunity and render non-responding tumors into responding tumors bear tremendous therapeutic potential. Accordingly, cumulative evidence suggests that antiviral innate immune responses hold intrinsic anticancer benefits by promoting antitumor immunity and thereby increasing efficacy of chemotherapy and immunotherapy (11,19–23).

In this study, we discovered that the top associated SNP rs13074711 regulates *TNFSF10* expression in TNBC cells, through the modulation of c-MYB binding activity at the motif. Furthermore, TNFSF10/TRAIL plays a significant role in regulating antiviral and antitumor immunity in TNBC. Our data suggest that genetic variants that drive aberrant expression of *TNFSF10* may contribute to antitumor defense mechanism and immunotherapy responses in aggressive breast cancer, especially in women of African descent.

## Results

### Genotype-specific expression of TNFSF10 in TNBC cells from Black women

**Figure 1:**
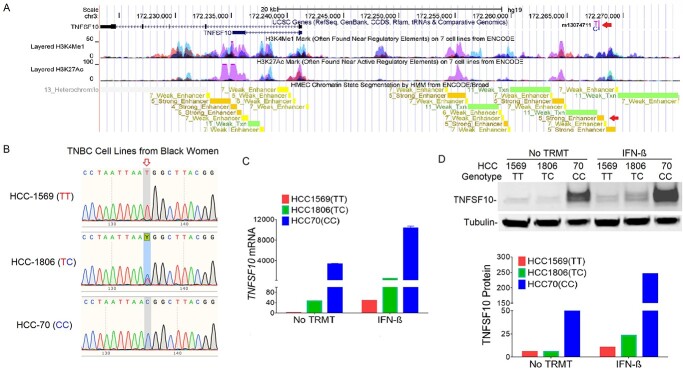
Genotype-specific expression of TNFSF10 in TNBC cell lines from Black women. (**A**) The UCSC Genome Browser shows the locations of rs13074711 and *TNFSF10*, along with ENCODE histone modification marks for H3K4Me1 and H3K27Ac of seven cell types and enhancer marks in HMEC. Arrows indicate the location of rs13074711 and a strong enhancer mark in HMEC. (**B**) DNA sequencing chromatograms are shown in three TNBC cell lines (HCC1569, HCC1806 and HCC70) from Black women. The arrow indicates the position of rs31074711 and Y represents TC heterozygote. (**C**) Expression of *TNFSF10* mRNA was measured by qRT-PCR in three cell lines with or without IFN-β treatment (24 h). Results were normalized to *RNA18S* and fold induction is shown relative to untreated HCC1569 cells. (**D**) Representative images (top) and a summary graph (bottom) of western blot analysis of TNFSF10 protein expression in three cells with or without IFN-β treatment. Tublin was used as an endogenous loading control. All data represent the mean and SD of *n* = 3–4 biological replicates and are representative of at least two independent experiments.

The top associated SNP rs13074711 is located 26.5 kb upstream of *TNFSF10* (*TRAIL*) in the region of high histone H3 lysine 4 monomethylation (H3K4Me1) and histone H3 lysine 27 acetylation (H3K27Ac) modifications (Fig. 1A). Strong enhancer features of the SNP were also found in human mammary epithelial cells (HMECs), suggesting that the association between variants in the 3q26.21 region and breast cancer risk may be mediated by regulating *TNFSF10* gene expression through the enhancer activities.

To evaluate the role of SNP rs13074711 in regulating TNFSF10 expression, we genotyped TNBC cell lines from Black women (Fig. 1B) and tested the correlation of rs13074711 genotypes with TNFSF10 expression (Fig. 1C and D). HCC-1569 cells with the TT genotype showed the lowest levels of mRNA (Fig. 1C) and protein expression (Fig. 1D), whereas those with the CC genotype showed the highest levels. Cells with the heterozygous genotype (TC) exhibited an expression level in-between homozygous (TT or CC)-genotype cells. Furthermore, the increased TNFSF10 expression in response to interferon (IFN)-β treatment is also genotype-dependent (the highest in CC-genotype cells and the lowest in TT-genotype cells). The results suggest that the SNP rs13074711 regulates TNFSF10 expression in TNBC cells from Black women.

### CRISPR-Cas9 editing of rs13074711 alters TNFSF10 expression and IFN-β-induced apoptosis

**Figure 2:**
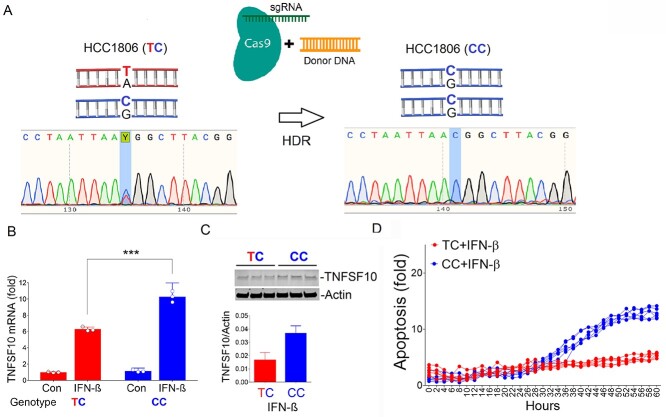
Regulation of TNFSF10 expression by rs31074711 Alleles. (**A**) Depicted is CRISPR-Cas9 genome editing of rs13074711 from TC genotype to CC genotype through HDR. DNA sequencing chromatograms showed the shift from T to C, after genome editing in HCC1806 cells. (**B**) qRT-PCR analysis of *TNFSF10* mRNA in HCC1806 cells with TC or CC genotype of rs31074711 that were treated with IFN-β for 1 day. Results were normalized to *RNA18S* and fold induction is shown relative to the untreated control with TC genotype. (**C**) Western blot analysis of TNFSF10 protein expression in HCC1806 cells with TC or CC genotype 1-day post IFN-β treatment. Tublin was used as an endogenous loading control. (**D**) HCC1806 cells with TC or CC genotypes were treated with IFN-β and subjected to the IncuCyte Live-Cell Imaging System. Apoptosis was quantified using green fluorescent signals from caspase-3/7–positive apoptotic cells normalized to cell density. All data represent the mean and SD of *n* = 3–6 biological replicates and are representative of at least two independent experiments. ^*^^*^^*^P < 0.001.

To directly test if rs13074711 genotypes have a significant effect on TNFSF10 expression, we engineered the TC genotype to the CC genotype in HCC-1806 cells, using CRISPR-Cas9 genome editing tools (Fig. 2A). An allelic change from TC to CC increased mRNA (Fig. 2B) and protein expression (Fig. 2C) of TNFSF10 in HCC-1806 cells, especially in response to IFN-β treatment. Furthermore, IFN-β-derived apoptosis was significantly greater in cells with the CC genotype compared with those with the TC genotype at 60-h post-treatment (12.8 ± 0.8 vs. 5.4 ± 0.4-fold increase) (Fig. 2D). Together, the data clearly showed that an allelic change in rs13074711 alters *TNFSF10* expression and thereby affects IFN-β-induced apoptosis. In the expression quantitative locus (eQTL) analysis of breast tumors using data from The Cancer Genome Atlas (TCGA), we found that rs13074711 genotype was associated with *TNFSF10* expression (*P* = 0.01) but not with other immune-related genes on different chromosomes (e.g. *TNF*, *TLR3*, *IL8*, *DDX58*, *IFIH* and *OAS1*), supporting our premise of *cis*-regulation of *TNFSF10* expression by the SNP genotype.

### rs13074711 regulates c-MYB binding activity in the *TNFSF10* enhancer

**Figure 3:**
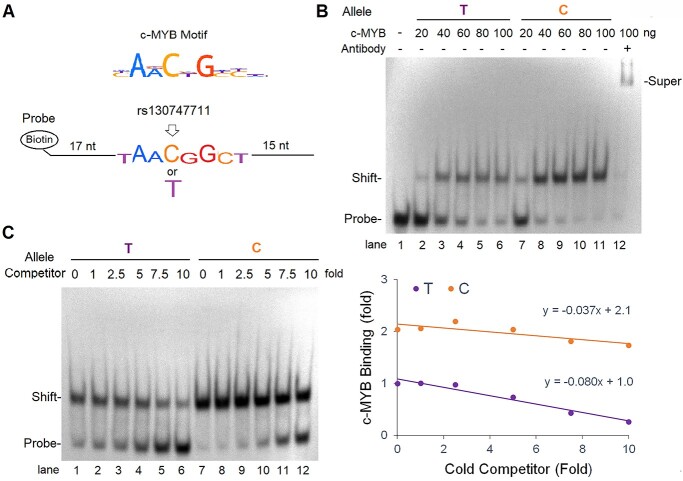
Regulation of c-MYB binding activity by rs13074711. (**A**) Depiction shows the c-MYB motif (top) and biotin-labeled oligonucleotides containing the *cis*-acting regulatory c-MYB element of the enhancer region (bottom). (**B**) EMSA was performed with biotin-labeled oligonucleotides (20 fmol) and recombinant c-MYB proteins (20–100 ng) with/without anti-MYB antibodies (200 ng). The mobilities of free oligonucleotides (probe), oligonucleotides associated with c-MYB (shift) or oligonucleotides associated both with c-MYB and anti-MYB antibodies (super-shift) were indicated. (**C**) Non-biotin-labeled oligonucleotides (cold competitor, 1–10-fold of hot probes) were incubated with c-MYB proteins (100 ng) before incubation with biotin-labeled probes (left). The densities of shift bands were plotted against the amounts of cold competitors added (right). Data are representative of at least three independent experiments.

To determine if rs13074711 controls *TNFSF10* expression through the regulation of enhancer activity, we conducted *in silico* analyses of *cis*-regulatory elements (motifs) of transcription factors. Our analysis predicts that an allelic change from T to C in rs13074711 may change the binding activity of c-MYB transcription factor at the motif (top, Fig. 3A). c-MYB is known to facilitate histone acetylation at super enhancer regions (24,25) and control cell proliferation, differentiation and apoptosis in many cell types (26). We thus examined whether rs13074711 regulates the enhancer activity of *TNFSF10* through the c-MYB transcription factor, by conducting electrophoretic mobility shift assay (EMSA) using biotin-labeled oligonucleotides with the T or C allele (bottom, Fig. 3A). A stronger association of c-MYB was observed at the C allele of rs13074711 (lane 7–11, Fig. 3B) compared with the T allele (lane 2 to 6, Fig. 3B), for all concentrations (20–100 ng) of c-MYB protein treatments. The specificity of c-MYB’s interaction with the motif was confirmed by further retardation in mobility (super-shift), resulting from an interaction with anti-c-MYB antibodies (lane 12, Fig. 3B). Furthermore, when we added increasing amounts of cold competitors (non-biotin-labeled oligonucleotides, 1- to 10-fold of biotin-labeled probes) to the reaction, the strength of this interaction decreased in a dose-dependent manner because of its competition with non-biotin-labeled oligonucleotides (left, Fig. 3C). Notably, the decrease in the interaction was more rapid in the T allele compared with the C allele (slope − 0.080 vs. − 0.037) (right, Fig. 3C). These data indicate that the C allele of rs13074711 strengthens the binding site for c-MYB transcription factor at the *TNFSF10* enhancer and thereby enhances *TNFSF10* expression (Fig. 2).

### *TNFSF10* expression in breast tumors and other tumors

**Figure 4:**
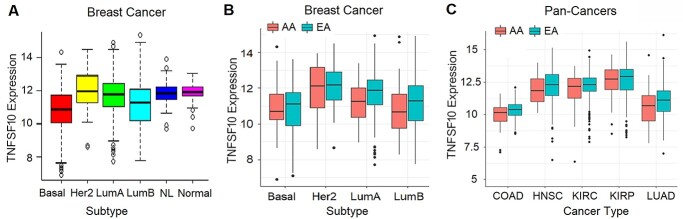
Comparison of *TNFSF10* expression between EA and AA in breast and other tumors. *TNFSF10* expression in the pan-cancer RNA-seq dataset was analyzed by (**A**) molecular subtypes of breast tumors (*n* = 1060); (**B**) by ethnicity and molecular subtypes of breast tumors; and (**C**) by ethnicity in five different types of tumors. The values are presented as log_2_ FPKM. Basal, basal-like; Her2, Her2-enriched, LumA, luminal A; LumB; NL, normal-like subtype of breast cancer. Normal indicates normal breast tissues. COAD, colon adenocarcinoma (*n* = 272); HNSC, head and neck squamous cell carcinoma (*n* = 492); KIRC, kidney renal clear cell carcinoma (*n* = 518); KIRP, kidney renal papillary cell carcinoma (*n* = 267); LUAD, lung adenocarcinoma (*n* = 440).

We next investigated TNFSF10 expression in patient samples using TCGA pan-cancer datasets (Fig. 4). When we analyzed the expression in breast tumors according to the molecular subtypes of breast cancer, we observed a strong association of the subtype with TNFSF10 expression in the multivariable model (*P* < 2.2 × 10−16) (Fig. 4A). Notably, the basal-like subtype had the lowest TNFSF10 expression among all subtypes, followed by the luminal B and luminal A subtypes.

Next, we examined the association of *TNFSF10* expression with ethnicity, after adjusting the subtype and age in the multiple linear regression. Race was modestly but significantly associated with *TNFSF10* expression (*P* = 0.03). On average, *TNFSF10* expression in breast cancer was 25% lower (0.4 unit in log2 scale) in AA compared with EA. Ethnic comparisons in each subtype of breast cancer showed that *TNFSF10* expression was significantly lower in AA than EA in the luminal A subtype, with a general trend of lower expression in AA in the basal-like and luminal B subtypes (Fig. 4B).

For other tumors, we focused on six cancer types, which included at least 40 AA patient samples in TCGA pan-cancer dataset (Fig. 4C). *TNFSF10* expression is ubiquitously lower in AA patients compared with EA patients in five types of cancers, with statistically significantly lower expression in AA in two types of cancers (colon and kidney cancers). Collectively, our data suggested reduced expression of *TNFSF10* in AA patients, which may contribute to resistance to *TNFSF10*-driven apoptosis of cancer cells and poor clinical outcomes.

### Correlation of *TNFSF10* expression with antiviral immune genes in TNBC tumors

**Figure 5:**
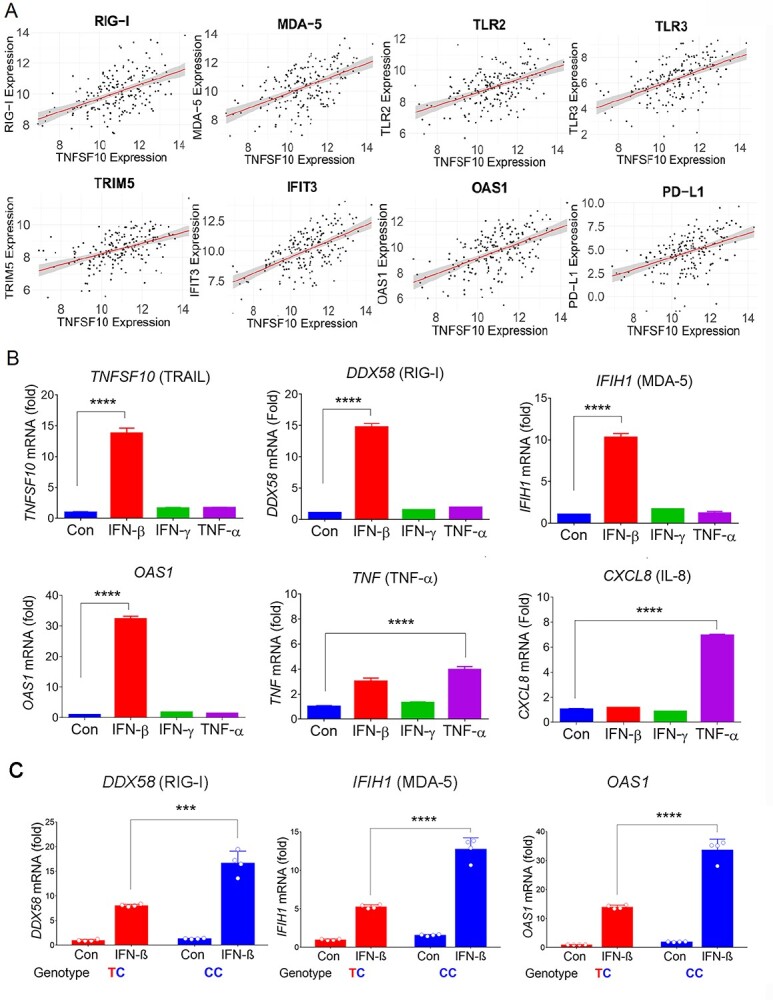
Correlation of *TNFSF10* expression with anti-viral gene expression and regulation by cytokines. (**A**) Examples of key immune genes significantly correlated with *TNFSF10* expression. Normalized RNA-seq data from TCGA-BRCA (*n* = 1076) were analyzed for correlation. The values are presented as log_2_ FPKM. (**B**) qRT-PCR analyses of *TNFSF10, DDX58, IFIH1, OAS1, TNF, IL* mRNA in HCC1806 cells that were treated with IFN-β, IFN-γ, or TNF-α for 1 day. (**C**) qRT-PCR analyses of *DDX58, IFIH1, OAS1* mRNA in HCC1806 cells with TC or CC genotype of rs31074711 that were treated with IFN-β for 1 day. Results were normalized to *RNA18S* and fold induction is shown relative to untreated control cells. Data represent the mean and SD of *n* = 3–4 biological replicates and are representative of at least two independent experiments. ^*^^*^^*^P < 0.001; ^*^^*^^*^^*^P < 0.0001.

To identify genes and pathways co-regulated with *TNFSF10*, we next conducted genome-wide correlation analyses in breast cancer using TCGA datasets. *TNFSF1*0 expression was significantly correlated with expression of 782 genes in basal-like breast cancer (*P* < 2.51 × 10^−6^) (Supplementary Material, Table S1), including significant enrichment of genes in immunogenic pathways and innate antiviral immune responses (Supplementary Material, Table S2). Examination of the correlated genes involved in innate immunity pathways revealed associations with known families of antiviral immunity genes, such as the RIG-I-like receptor signaling family and Toll-like receptor (TLR) signaling genes (Fig. 5A) (Supplementary Material, Fig. S1). These genes play important roles in stimulating type 1 IFN production and recognizing viral dsRNA to provide resistance to most RNA viruses. Other antiviral gene families identified from the GO term analysis included the tripartite motif family involved in viral infection and replication restriction, interferon-stimulating gene family, IFN-induced proteins with tetratricopeptide repeats (IFIT) and the oligoadenylate synthase (OAS) family, which is involved in viral RNA degradation.

The pathway analysis also identified a high-fold enrichment of several other inflammatory and adaptive immunity pathways from the gene set that included cytokine signaling processes, various processes of T-cell chemotaxis and proliferation, as well as MHC Class I antigen processing and presentation (Supplementary Material, Fig. S2). Notably, *TNFSF10* expression had an additional highly significant correlation with expression of *CD274* (PD-L1) (Fig. 5A), a therapeutic target and predictive biomarker in cancer immunotherapy.

### *TNFSF10* expression is induced by type I IFN, not by TNF-α

Because *TNFSF10* expression is correlated with expression of antiviral immune genes in TNBC tumors, we assessed whether *TNFSF10* expression is regulated by type I IFNs in TNBC cells (Fig. 5B). IFN-β dramatically increased *TNFSF10* expression in TNBC cells, along with expressions of other classical antiviral genes [*DDX58* (RIG-I), *IFIH1* (MDA-5) and *OAS1*]. In contrast, *TNFSF10* expression was minimally induced by IFN-γ or TNF-α treatment, which significantly stimulated *TNF* (TNF-α) and *CXCL8* (IL-8) expression. Collectively, the data indicated that, although *TNFSF10* is a member of TNF superfamily, its expression is not regulated by TNF-α-mediated inflammatory signaling, but rather by type I IFN-mediated innate immune signaling.

### Correlation of antiviral gene expression with SNP rs13074711 genotypes

We next examined the relationship between antiviral gene expression and SNP genotypes in TNBC cells (Fig. 5C). This is partly because *TNFSF10* expression is correlated with expression of antiviral immune genes, induced by IFN-β, and is also regulated by SNP rs13074711. We utilized CRISPR-Cas9 edited HCC-1806 cells with the TC or CC genotypes of rs13074711, which were correlated with *TNFSF10* expression (Fig. 2). An allelic change from TC to CC significantly increased mRNA expression of antiviral genes [*DDX58* (RIG-I), *IFIH1* (MDA-5) and *OAS1*], especially in response to IFN-β treatment. Expression of RIG-I (*DDX58*) and MDA-5 (*IFIH1*) was 2.07-fold and 2.44-fold higher, respectively, in cells with the CC genotype compared with those with the TC genotype after IFN-β treatment. The data indicate the importance of the SNP in regulating *TNFSF10* expression, which may subsequently contribute to antiviral immune responses in TNBC cells.

### TNFSF10 plays an essential role in poly(I:C) or IFN-β-driven apoptosis

**Figure 6:**
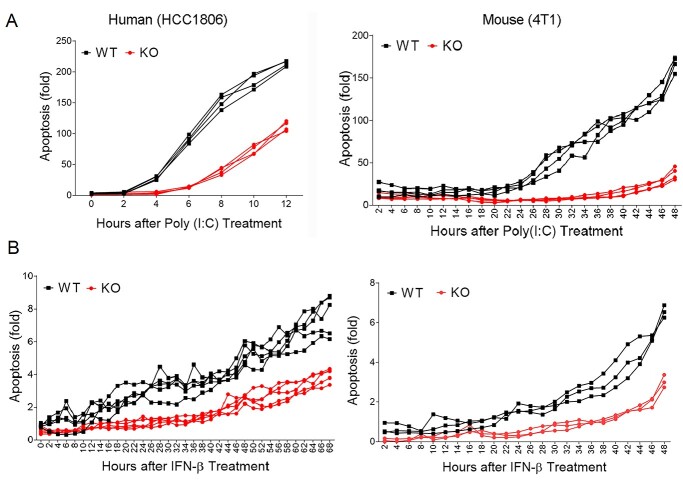
Decreased apoptosis of *TNFSF10-*KO cells in response to poly(I:C) and IFN-β. (**A** and **B**) Apoptosis of *TNFSF10*-WT and KO cells was analyzed using the IncuCyte Live-Cell Imaging System after poly(I:C) (A) or IFN-β (B) treatment. Apoptosis was quantified using green fluorescent signals from caspase-3/7-positive apoptotic cells normalized to cell density. Data represent the mean and SD of *n* = 4–10 biological replicates and are representative of at least two independent experiments.

To investigate the role of TNFSF10 in regulating antiviral defense mechanism, we utilized CRISPR-Cas9 technology and deleted the *TNFSF10* gene from TNBC cells (Supplementary Material, Fig. S3). Deletion of *TNFSF10* did not change proliferation (Supplementary Material, Fig. S3C) or apoptosis (Supplementary Material, Fig. S3D), before stimulation of antiviral immune responses. However, when we treated cells with poly(I:C), a synthetic analogue of double-stranded RNA virus, we observed a clear difference in poly(I:C)-induced apoptosis between the wild-type (WT) and *TNFSF10-*KO cells from human (HCC1806) and mouse (4 T1) TNBC cell lines (Fig. 6A). Depletion of *TNFSF10* clearly reduced poly (I:C)-induced apoptosis, while poly (I:C) treatment led to a dramatic increase in apoptosis of WT cells. Similarly, loss of *TNFSF10* clearly reduced IFN-β-induced apoptosis, while IFN-β treatment significantly induced apoptosis of WT cells (Fig. 6B). Collectively, our data suggested that TNFSF10 plays an essential role in antiviral immunity-induced apoptosis in TNBC cells, partly through type I IFN signaling.

### Loss of *TNFSF10* inhibits T-cell infiltration into tumors, *in vivo*

**Figure 7:**
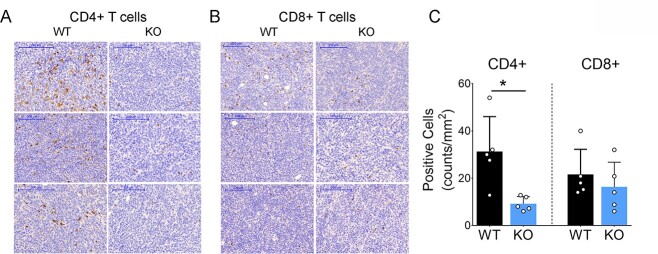
Decreased infiltration of CD4+ T cells into *TNFSF10*-KO breast tumors, *in vivo*. (**A** and **B**) Representative images from IHC analyses of CD4+ or CD8+ T cells in 4 T1 breast tumors with *TNFSF10*-KO or WT genotypes engrafted into syngeneic mice. Tumor-infiltrating CD4+ or CD8+ T cells were stained with anti-mouse CD4 or anti-mouse CD8 antibodies (in brown) and compared between *TNFSF10*-KO and WT tumors. Original magnification of 200× was used for scoring. The scale bar of 200 μm was included in all images. (B) The density (number) of CD4 or CD8 positive cells was estimated by counting all immuno positive cells in five high-powered fields. The result was presented as a number of positively stained cells per 1 mm^2^ of each tumor (the mean and SD) from five tumors (^*^^*^P < 0.01).

Lastly, we investigated the effect of *TNFSF10*-deficient tumor cells in regulating immune cell infiltration and antitumor immunity, using an immunocompetent syngeneic mouse model of 4 T1 breast cancer (Fig. 7). Tumor cells with *TNFSF10*-KO or WT genotypes were engrafted into BALB/c mice, monitored for tumor growth for 27 days and harvested for immune staining at the termination of the mice. We did not observe significant differences in tumor growth between mice with *TNFSF10*-KO or WT tumors, consistent with our *in vitro* data showing no significant difference in proliferation between *TNFSF10*-KO and WT cells before stimulation of antiviral immune responses (Supplementary Material, Fig. S3C). However, there were clear differences in the levels of tumor-infiltrating CD4+ or CD8+ T cells between *TNFSF10*-KO and WT tumors (Fig. 7A and B), with adjacent lymph nodes used as positive controls for CD4 and CD8 staining (Supplementary Material, Fig. S4). The mean number of CD4+ cells in the WT group was 31.3 ± 6.6 (counts/mm^2^), which was dramatically reduced to 9.2 ± 1.3 in the knockout (KO) group (Fig. 7C). The average number of CD8+ cells was modestly reduced from 21.6 ± 4.7 in the WT group to 16.3 ± 4.6 in the KO group. These data together indicate an important role for TNFSF10 in regulating the TME and TILs, suggesting that reduction of TNFSF10 expression may contribute to an unfavorable TME and decreased sensitivity to chemotherapy and immunotherapy.

## Discussion

Following our previous identification of genetic variants associated with ER-negative breast cancer in women of African descent (5), we investigated the molecular mechanisms of SNP influences on gene regulation and tumor progression in TNBC. The top SNP rs13074711 resided in the enhancer region of *TNFSF10*/*TRAIL* (Fig. 1) and regulated *TNFSF10* expression in TNBC cells from Black women (Fig. 2). *In silico* analyses followed by experimental validation (EMSA) showed that rs13074711 regulated *TNFSF10* expression through c-MYB activity at the enhancer (Fig. 3). Furthermore, comparison of *TNFSF10* expression between AA and EA women revealed relatively lower expression of *TNFSF10* in AA in breast tumors and other tumors (Fig. 4). Interestingly, *TNFSF10* expression was significantly correlated with the expression of antiviral immune genes and was regulated by type I IFN, not by TNF, in TNBC cells (Fig. 5). Accordingly, CRISPR-Cas9 KO of *TNFSF10* led to a profound decrease in apoptosis of TNBC cells in response to poly(I:C) and IFN-β (Fig. 6). Lastly, *in vivo* experiments showed that *TNFSF10*-deficient tumors reduced the levels of tumor-infiltrating CD4+ and CD8+ T cells (Fig. 7), and thereby regulated the TME and antitumor immunity. Collectively, our data showed that a genetic variant associated with breast cancer in women of African descent regulated *TNFSF10* expression in TNBC, which plays a significant role in the regulation of antiviral and antitumor immune responses. Our results may help to understand the roles of genetic factors in the regulation of immune defense mechanism and to improve therapeutic options for TNBC patients, especially women of African ancestry.

It is worth emphasizing that our understanding of the genetic risk factors for human complex diseases remains limited, partly because of our incomplete understanding of the significance of non-coding regions in the human genome. The haploid human genome occupies a total of over 3 billion DNA base pairs, but only about 1.5% of the genome codes for proteins, far fewer than had been expected before its sequencing. The rest of the human genome consists of non-protein-coding sequences with unknown biologic functions. Because the majority of common risk alleles discovered through GWAS map to non-protein-coding regions, it has been challenging to determine the biological functionality of SNPs in the development of human diseases.

In this study, we applied CRISPR-Cas9 genome-editing tools to specifically edit rs13074711, which resides in a non-protein-coding region of the human genome and identified the biological role of rs31074711 in regulating *TNFSF10* expression in breast cancer. We also conducted *cis*- and *trans*-eQTL analyses of breast tumors using data from TCGA, confirming a significant association of rs13074711 genotypes with *TNFSF10* expression (*P* = 0.01) (through *cis*-eQTL analyses), but not with other immune-related genes on different chromosomes (*trans*-eQTL analyses). Considering that *trans*-eQTL identification is much more challenging than *cis*-eQTL because a greater number of SNP-gene pairs are tested for trans-association on different chromosomes (27), further studies with much larger sample sizes or new methodologies are warranted to test the SNP association with other genes on different chromosomes. Nevertheless, our study suggests that rs13074711 regulates c-MYB binding activity and thereby controls *TNFSF10* expression. Although the signaling pathways of c-MYB in the regulation of *TNFSF10* expression merit further investigation, our results revealed an unidentified role for the non-coding SNP rs31074711 in the regulation of gene expression and immune defense mechanism in TNBC cells.

Interestingly, despite the fact that *TNFSF10* is one of the TNF superfamily members, its expression was induced by type I IFNs but not by TNF in TNBC cells. In support of this, previous findings showed the regulation of *TNFSF10* expression by IFNα in blood cells (28,29). Furthermore, our data clearly showed that *TNFSF10* expression was highly correlated with antiviral gene expression in TNBC tumors, rather than with inflammatory or apoptosis-related gene expression. More importantly, when we treated breast cancer cells with poly(I:C), a synthetic analogue of double-stranded RNA virus, depletion of *TNFSF10* clearly reduced poly(I:C)-induced apoptosis, compared with WT cells which underwent severe apoptosis upon poly(I:C) treatment (Fig. 6). Our experimental results, along with TCGA data analyses, suggested that TNFSF10 may play a significant role in antiviral defense mechanisms in TNBC cells.

Although TRAIL’s capacity for inducing cancer cell death and clearance of virus infection has been investigated in depth (30,31), there is still limited knowledge regarding whether and to what extent TNFSF10/TRAIL signaling can affect the antiviral response and immune tumor environment in cancer cells, especially TNBC cells. This is partly because most mice models used for tumor xenografts were athymic nude mice or immune-deficient NOD/SCID mice (32,33), which were unable to produce most T cells and were unable to exhibit the actual TME. We thus utilized the immunocompetent 4 T1 syngeneic mouse model and determined the effects of *TNFSF10*-KO on regulation of T-cell infiltration in breast tumors. We found that tumor-infiltrating CD4+ T cells were significantly reduced in *TNFSF10*-KO tumors compared with the WT tumors, indicating that TNFSF10 could regulate immune cell infiltration of tumors and thereby TILs in the TME. Considering that tumors with a high density of TILs in the TME are more likely to respond to immune checkpoint inhibitors (16–18), our data suggest that TNBC tumors with dysregulated TNFSF10 expression might have a reduced sensitivity to immune checkpoint inhibitors, which warrants further investigation.

To our knowledge, this is the first functional study to identify genetic and molecular mechanism of SNPs associated with aggressive breast cancer in women of African ancestry. It is unfortunate that there remain huge gaps in our knowledge of the molecular pathways that lead to aggressive breast cancer in Black women, who have high prevalence of ER-negative breast cancer and the associated worse clinical outcomes. Our results revealed a novel role for the non-coding SNP rs31074711 in the regulation of *TNFSF10* expression and immune defense responses in TNBC cells. We also identified the mechanism of rs31074711-regulated *TNFSF10* expression, which involves the modulation of c-MYB binding activity at the motif. Furthermore, we identified consistently lower expression of *TNFSF10* in many cancers of AA compared with EA patients. Lastly, our data from *in vivo* experiments demonstrated an important role for TNFSF10 in regulating immune cell infiltration of tumors in the TME of TNBC tumors. As increasing evidence shows that antiviral innate immune responses promote antitumor immunity and the efficacy of chemotherapy and immunotherapy (11,19–23), our results may increase understanding of breast cancer susceptibility genes that can influence cancer development/progression through the regulation of immune defense mechanisms. Better understanding of the roles of genetic variants in the regulation of tumor immune microenvironment may also improve therapeutic options for TNBC patients, especially women of African ancestry.

## Materials and Methods

### Animal study and handling

All animals were humanely handled and monitored for health conditions according to the Institutional Animal Care and Use Committee approved protocols. Eight-week-old female BALB/c mice (Charles River Laboratories, Wilmington, MA) were anesthetized via inhalation with 2% vaporized isoflurane and were injected with 1 × 10^5^ 4 T1 breast cancer cells (100 μl, 50% Matrigel) into the fourth inguinal mammary gland at the base of the nipple. Tumor measurements were performed weekly using calipers to calculate tumor volume using the formula: 1/2 (length × width^2^). The assessment was blind to the animal groups and performed consistently throughout the study. Mice were sacrificed at the end of the study (27 days after injection) and tumors were collected from animals in each group for downstream analysis.

### Immunohistochemistry (IHC) of mouse tumors

Tumors collected from both study groups (*n* = 5, each) were fixed in 10% formalin for 24 h, dehydrated in 70% ethanol and embedded in paraffin. Five micrometer sections from each Formalin-Fixed Paraffin-Embedded (FFPE) block were used for H&E, anti-CD4 and anti-CD8 staining. The IHC staining was performed according to the standard protocol on Leica Bond RX automated Stainer. After deparaffinization and rehydration, tissue sections were treated with Target Retrieval Solution (Leica Biosystems, #AR9640) for heat treatment for 20 min. Anti-mouse CD4 antibody (Synaptic Systems, HistoSur #HS-360-117, Clone: 78H9D2, 1:200 dilution) and anti-mouse CD8 antibody (eBioscience Affymetrix, #14–0808-82, Clone: 4SM15, 1:800 dilution) were applied to tissue sections for 1-h incubation at room temperature. The antigen–antibody binding was detected by ImmPress anti-rat IgG Polymer-HRP kit (Vector Laboratories, #MP-7444) and DAB (Leica Biosystems, Bond Polymer Refine kit). The slides stained for CD4+ and CD8+ were digitized on a 3D HISTECH pannoramic whole slide scanner. The density (number) of CD4 or CD8-positive cells was estimated by counting all immune-positive cells in five high-powered fields (1 mm^2^ each). The scoring and calculation of an averaging score were conducted by a pathologist (GFK), as previously described (34,35). The result was presented as the number of positively stained cells per 1 mm^2^.

### Cell culture

Breast cancer cell lines from humans (HCC1569, HCC1806 and HCC70) and mice (4 T1) were obtained from the American Type Culture Collection (ATCC, Manassas, VA). Cells were authenticated for species and unique DNA profile using short tandem repeat analysis by the provider. Cells were cultured in media recommended by ATCC and tested for mycoplasma contamination using the MycoAlert kit (Lonza, #LT07–318).

### CRISPR-Cas9 knockout (KO) and genome editing

The KO experiment was performed as previously described with modifications (36). Briefly, a pair of gRNAs (crRNAs and tracrRNAs) was designed to delete 16 693 bp of *TNFSF10* sequence using Alt-R CRISPR-Cas9 guide RNA tools (Integrated DNA Technologies, IDT), and was synthesized by the company. After incubation with S.p.Cas9 Nuclease V3 (IDT, #1081058), the gRNAs were transfected into TNBC cells according to the manufacturer’s instructions. For genome editing, gRNAs were designed to target the *TNFSF10* enhancer region containing SNP rs13074711, which were incubated with S.p.Cas9 Nuclease V3 and transfected into TNBC cells, along with Alt-R homology-directed repair (HDR) donor oligos (IDT) for homologous recombination. DNA sequences of gRNAs or donor oligos will be provided upon request.

### qRT-PCR and western blot analyses

qRT-PCR was performed using the TaqMan Gene Expression Master Mix (Thermo Fisher Scientific, #4369016), along with TaqMan Assays for *TNFSF10* (Thermo Fisher Scientific, Hs00921974_m1), *DDX58* (IDT, Hs.PT.58.4273674), *IFIH1* (IDT, Hs.PT.58.1224165), *OAS1* (IDT, Hs.PT.58.2338899), *TNF* (IDT, Hs.PT.5845380900) or *CXCL8* (IDT, Hs.PT.5839926886). The fold change in expression of each gene was calculated using the ΔΔCT method, with *RNA18S* (IDT, Hs.PT.39a.22214856.g) as an internal control. To detect protein expression of TNFSF10, we conducted western blot analyses as previously described (36), using anti-TNFSF10 (Cell Signaling, #3219, 1:300 dilution), anti-tubulin (Cell Signaling, #2128, 1:2000 dilution) and anti-β-actin (abcam, #ab8227, 1:10 000 dilution) antibodies. After incubating with a fluorescent secondary antibody (Li-Cor, #926–68 071, 1:20 000 dilution), the fluorescence signals were detected by Li-Cor Odyssey Classic Infrared Imaging System, according to the manufacturer’s protocol.

### The IncuCyte live-cell proliferation and apoptosis analysis

Cells plated at 5000 cells/well for HCC1806 (and 4000 cells/well for 4 T1) in 96-well plates were transfected with poly(I:C)-LMW (InvivoGen, #tlrl-picw) using Lipofectamin 3000 (Thermo Fisher Scientific, #L3000015). We used 50 ng and 200 ng of poly(I:C)-LMW for HCC1806 and 4 T1 cells, respectively. For cytokine treatment, HCC1806 cells were treated with 100 units of recombinant human IFN-beta (IFN-β) 1a protein (R&D systems, #11415–1), whereas mouse 4 T1 cells were treated with 2 ng of recombinant mouse IFN-β protein (R&D Systems, #8234-MB-010). Images of live cells were acquired using a 10× objective every 2 h (4 images/well) with the IncuCyte® S3 Live-Cell Analysis System, as described previously (36).

### EMSAs

The construct expressing c-MYB was generously provided by Dr Odd Gabrielsen (University of Oslo, Norway) and recombinant c-MYB proteins were purified following his protocol (37). The *cis*-acting regulatory element of c-MYB on the *TNFSF10* enhancer was analyzed using ENCODE ChIP-seq data from the UCSC Genome Browser and the consensus sequences were identified by TF finder (http://tfbind.hgc.jp). On the basis of sequences, two biotin-labeled synthetic oligonucleotides were synthesized with the T or C allele (IDT) and annealed together. Twenty to 200 fmols of oligonucleotides were incubated with 20–200 ng of recombinant c-MYB proteins, 1–10 fmols of oligonucleotides or 100–200 ng of anti-c-MYB antibodies (Cell Signaling, #12319) in the presence of 2.5% glycerol, 5 mM MgCl_2_ and 0.05% NP-40 using the LightShift Chemiluminescent EMSA kit (ThermoFisher, #20148, as previously described (36).

### TCGA data analysis

TCGA pan-cancer RNA-seq and clinical data were used to examine the distributions of *TNFSF10* expression across different subtypes of TCGA breast invasive carcinoma (TCGA-BRCA) and across different cancer types. First, we analyzed ethnic differences in *TNFSF10* expression in TCGA-BRCA, using linear regressions after adjusting the age at diagnosis. We also examined racial differences in TNFSF10 expression in six other common cancers with at least 40 AA patients. Second, we conducted a correlation analysis of gene expression in female breast cancer patients with the ‘Basal’ PAM50 subtype for *TNFSF10* expression with all other genes (*n* = 19 929) using the Pearson correlation method. An empirical *P* < 2.51 × 10^−6^ was considered statistically significant after correcting for multiple testing by the Bonferroni method (0.05/19929). To identify enriched pathways and biological processes of genes significantly correlated to TNFSF10, pathway analysis of 782 identified genes was performed using the DAVID’s Functional Annotation Tool (https://david.ncifcrf.gov/). Gene Ontology (GO) terms and BioCarta pathways were used for pathway analysis and functional annotation. GO terms were further filtered for redundant and obsolete terms with the Revigo tool (http://revigo.irb.hr/) at medium (0.7) setting. We also conducted eQTL analysis using breast tumors from TCGA, as described previously (38). Briefly, we examined an association between the SNP rs13074711 with the expression of *TNFSF10* (*cis*-eQTL) or other immune-related genes on different chromosomes (*trans*-eQTL). Age at diagnosis, molecular subtype, race and copy number change were adjusted for in the linear regression models.

### Statistical analysis of experimental data

Each experiment was repeated at least three times. The mean ± standard deviation (SD) of TNFSF10 mRNA or protein expression is presented and compared between two groups using the unpaired *t*-test and between three or more groups using the one-way ANOVA. Statistical significance was set at ^*^P < 0.05; ^*^^*^P < 0.01; ^*^^*^^*^P < 0.001 and ^*^^*^^*^^*^P < 0.0001. Plots were generated using Graphpad Prism 6.0 (GraphPad Software, Inc., La Jolla, CA). Two-sided *P* values < 0.05 were considered statistically significance.

## Supplementary Material

20220420_Supplementary_Informations_Final_ddac168
