# Supplementary material for: An enhancer variant associated with breast cancer susceptibility in Black women regulates TNFSF10 expression and antitumor immunity in triple-negative breast cancer
Source: Hum Mol Genet. 2022 Aug 5;32(1):139–50. doi: 10.1093/hmg/ddac168 (PMC9837834; doi:10.1093/hmg/ddac168)
Supplement: 20220420_Supplementary_Informations_Final_ddac168 [file 20220420_supplementary_informations_final_ddac168.docx]

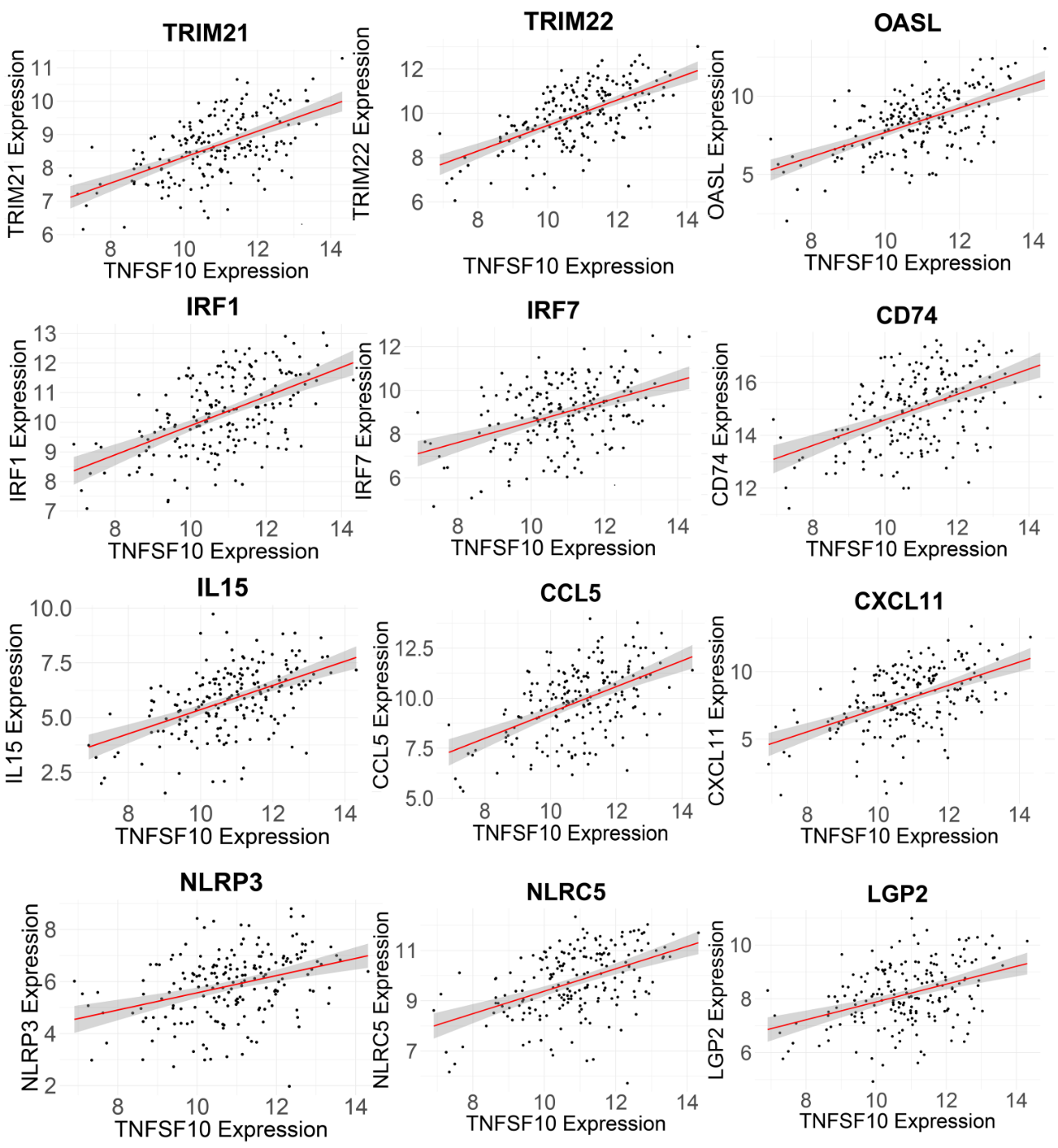


**Supplementary Fig. S1. Genes Correlated with *TNFSF10* Expression.** Additional examples of key anti-viral immune genes significantly correlated with *TNFSF10* expression, including genes related to tripartite motif family, NOD-like receptor, RIG-like receptor, interferon regulatory factor, and viral translation inhibitory immune pathways. Normalized RNA-seq data from TCGA-BRCA (*n*=1,076) were analyzed for correlation (*p*<2.51x10^-6^). The values are presented as log_2_ RSEM.

**Supplementary Fig. S2. GOterms of Correlated Genes**. Selection of significant GOterms of correlated genes to *TNFSF10* with the highest fold enrichments. Significantly correlated genes (*n*=782) were inputted into the DAVID tool and a list of GOterms were generated (significance threshold set at Bonferroni-corrected *p*<0.05). Obsolete and redundant GOterms were further removed by the Revigo tool using medium setting. Pathways related to T-cell proliferation and chemotaxis, antigen presentation, and various innate inflammatory processes were identified as highly enriched.

**
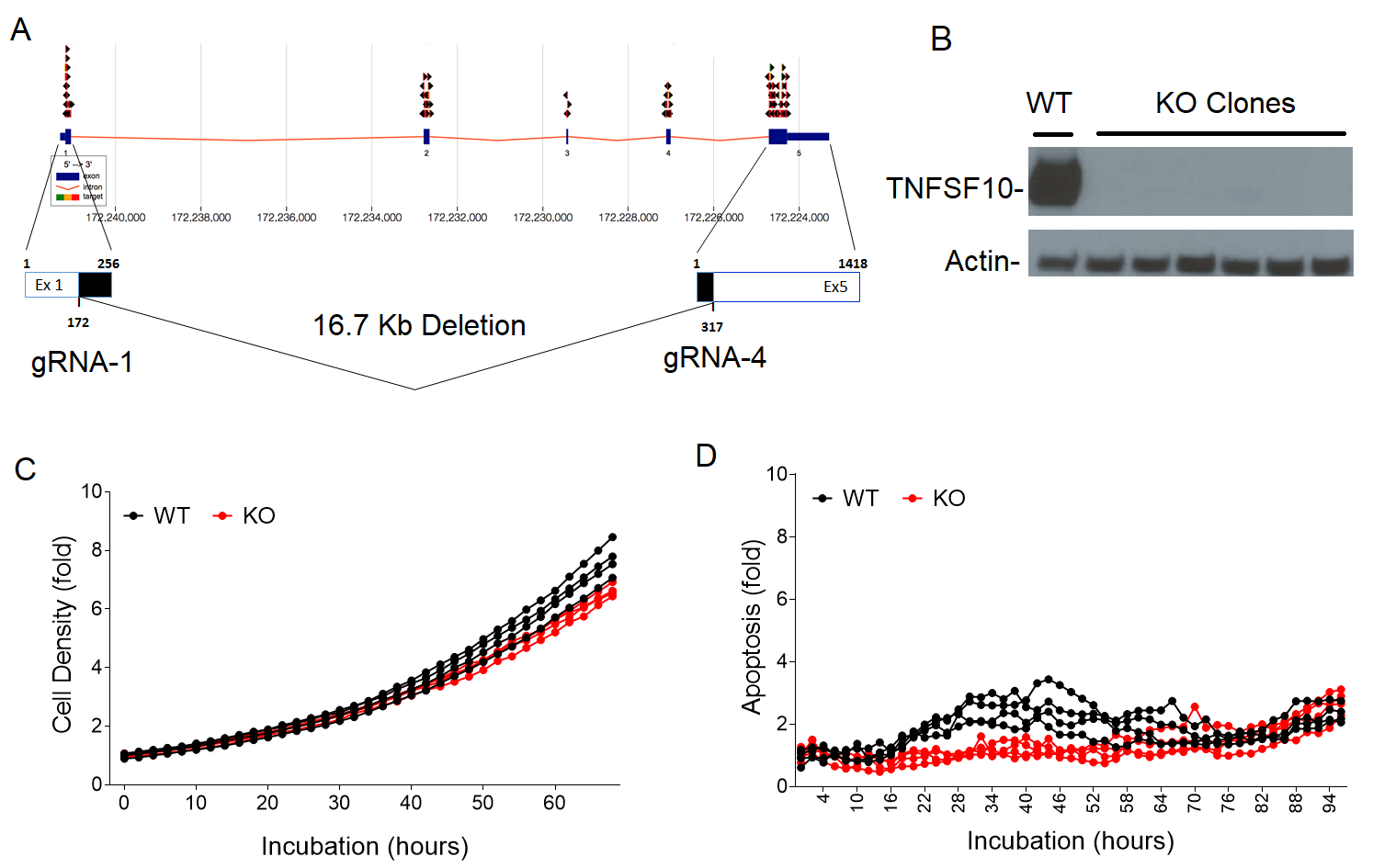
**

**Supplementary Fig. S3. Depletion of TNFSF10 from TNBC cell lines.** (A) Deletion of 16.7 Kb region of *TNFSF10* using CRISPR-Cas9 genome-editing tools. (B) No expression of *TRAIL* (*TNFSF10*) was confirmed in the knockout (KO) cells, while it was strongly expressed in the wild type (WT) cells, using Western blot analysis. Actin was used as an endogenous control. (C and D) Proliferation and apoptosis of *TNFSF10*-KO and WT cells was measured by IncuCyte live-cell imaging system. Apoptosis was quantified using green fluorescent signals from caspase-3/7–positive apoptotic cells normalized to cell density. Data represent mean and SD of *n*=4 to 10 biological replicates and are representative of at least two independent experiments.


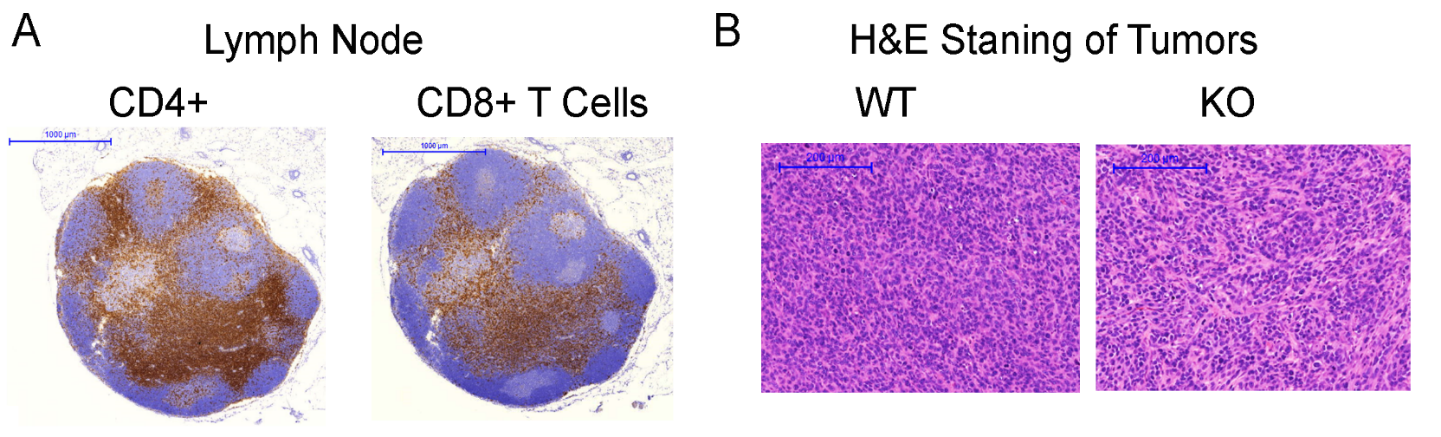


**Supplementary Fig. S4. Immunohistochemical staining of mouse breast tumors and lymph nodes.** (A) Tumor adjacent lymph nodes stained with anti-mouse CD4 and CD8 antibodies showed strong signals and were used as positive controls for the antibodies. A scale bar of 1000 μm was shown in each image. (B) Representative H&E staining images of TNFSF10-knockout (KO) and the wild type (WT) tumors. A scale bar of 200 μm was shown in each image.
